# Childhood Psychological Problems in School Settings in Rural Southern Africa

**DOI:** 10.1371/journal.pone.0065041

**Published:** 2013-06-12

**Authors:** Melissa A. Cortina, Mina Fazel, Tintswalo Mercy Hlungwani, Kathleen Kahn, Stephen Tollman, Mario Cortina-Borja, Alan Stein

**Affiliations:** 1 Oxford University Department of Psychiatry, Warneford Hospital, Oxford, United Kingdom; 2 MRC/Wits Rural Public Health and Health Transitions Unit, University of the Witwatersrand, Johannesburg, South Africa; 3 School of Public Health, University of the Witwatersrand, Johannesburg, South Africa; 4 MRC Centre of Epidemiology for Child Health, UCL Institute of Child Health, London, United Kingdom; Catholic University of Sacred Heart of Rome, Italy

## Abstract

**Background:**

Many children can be exposed to multiple adversities in low and middle-income countries (LMICs) placing them at potential risk of psychological problems. However, there is a paucity of research using large representative cohorts examining the psychological adjustment of children in school settings in these countries. Children’s psychological adjustment has been shown to affect educational progress which is critical for their future. This study, based in a rural, socio-economically disadvantaged area of South Africa, aimed to examine the prevalence of children’s psychological problems as well as possible risk and protective factors.

**Methods:**

Rates of psychological problems in 10–12 year olds were examined using teacher- and child-report questionnaires. Data on children from 10 rural primary schools, selected by stratified random sampling, were linked to individual and household data from the Agincourt health and socio-demographic surveillance system collected from households over 15 years.

**Results:**

A total of 1,025 children were assessed. Teachers identified high levels of behavioural and emotional problems (41%). Children reported lower, but substantial rates of anxiety/depression (14%), and significant post-traumatic stress symptoms (24%); almost a quarter felt unsafe in school. Risk factors included being a second-generation former refugee and being from a large household. Protective factors highlight the importance of maternal factors, such as being more educated and in a stable partnership.

**Conclusion:**

The high levels of psychological problems identified by teachers are a serious public health concern, as they are likely to impact negatively on children’s education, particularly given the large class sizes and limited resources in rural LMIC settings. Despite the high levels of risk, a proportion of children were managing well and research to understand resilience could inform interventions.

## Introduction

The importance of mental health as a key component in child and adolescent development is beginning to influence global health initiatives [1] and is a crucial component of the UN Millennium Development Goals [2]. This is of particular significance because it is predicted that when the current generation of children become adults, depression will be the second greatest contributor of disease globally [3]. For children growing up in socioeconomically disadvantaged areas, safe and supportive home and school environments are central to ensure they reach their potential [4] and maintain their psychological health [5].

Chronic adversity is one of the biggest risk factors for psychological problems in children [6]. In high-income countries, psychological problems have been shown to have a negative impact on a child’s ability to fulfil their educational and developmental potential [7]. A recent meta-analysis of epidemiological studies of child mental health in Southern Africa showed that rates of psychological problems in children and adolescents are around 20%, within a wide range (2.7–64.8%) [8]. A major limitation of that review was the small number of studies included and the lack of large representative cohorts. In particular, very little research has been conducted using large representative cohorts examining psychological adjustment and experiences of children or adolescents in school settings in low-income and middle-income countries (LMICs). This is important because children living in rural areas in LMICs are often exposed to multiple stressors [1]. Socioeconomic disadvantages, such as limited access to clean water, and high levels of HIV, malaria and other infections, exposes many children to illness and death in family members. Furthermore, many areas have received large numbers of displaced families due to war or for socioeconomic reasons. All these factors make children vulnerable to a spectrum of psychological problems [9], [10].

There is now a large body of evidence to suggest that education is crucial to the long-term well-being and future life opportunities of children, especially in socio-economically adverse circumstances in LMICs [11]. Children who repeat grades or do not complete school are at the greatest risk. Girls’ education is particularly important as many start to have children at relatively young ages in LMICs and education has a strong impact, not only on their own life chances, but on their respective children’s survival, health and development [12], [13]. The situation is made especially difficult because of poor resources resulting in large class sizes. One of the key predictors of educational success is emotional and behavioural adjustment [14]. Therefore, if there are substantial numbers of children with emotional and behavioural problems, this is likely to make the teacher’s task difficult, especially in larger classes, which may, in turn, affect the quality of education for all the children in that class. There is, however, a paucity of published studies of children’s emotional and behavioural adjustment in Southern Africa.

A substantial gap therefore exists in the evidence base in LMICs, despite the need to scale up services in the most resource-poor areas of the world [15]. The specific areas of need should be identified to direct the development of appropriate interventions, as highlighted by the WHO’s Mental Health Gap Action Programme [16]. An accurate identification of the nature and prevalence of mental health problems, as well as risk and protective factors, is an important starting point for reducing the burden of psychological disorders and promoting resilience [16], [17].

We focused on a large area in the north-east of South Africa near the Mozambique border. The research was conducted in this area for two main reasons: first, it represents a poor underdeveloped area of South Africa with limited resources and poor services [18], high rates of migration leading to family disruption, and high levels of infection especially HIV resulting in growing numbers of children dealing with chronic illness, and sometimes death in family members [19]. Second, the area has an established research infrastructure enabling the recruitment of a large representative cohort and on-going socio-demographic surveillance since 1992, which has facilitated the prospective collection of a range of risk and protective factors [20].

We thus examined a representative group of 10–12 year olds to determine the prevalence of behavioural and emotional problems, including post-traumatic stress symptoms; and to identify risk and protective factors from prospectively collected individual and household data. In addition to children’s self-report data, teacher-reported data was collected to provide an independent view of children’s psychological functioning. The age group, 10–12 years, was selected because psychological problems often emerge as puberty approaches and this may be an opportune time for intervention [21].

## Methods

### Ethics Statement

Research Ethics Approval was obtained from the Witwatersrand University Committee for Research on Human Subjects (Medical; M070221) and the Oxford Tropical Research Ethics Committee (008–07), the local Department of Education and each school’s Governing Body. Extensive discussions with local health, education and social welfare governmental organizations were undertaken during the development of this research to address questions and concerns about the study and obtain consent. Informed, written parental consent and written child assent were obtained.

### Study Site, Design and Participants

The research was conducted within the Agincourt health and socio-demographic surveillance site (Agincourt HDSS) in the sub-district of Mpumalanga Province in north-eastern South Africa bordering Mozambique [22]. Since 1992, some 16,000 households, in 27 contiguous villages, are interviewed on an annual basis to update key health, social and demographic variables. In the study site, only 57% of children and adolescents aged 6–15 live in a household with both parents present, with many fathers absent either permanently or temporarily for work [23]. A third of all deaths in the site are attributed to HIV [24]. This area has a considerable in-migrant population from Mozambique following its civil war from 1977–1992. One third of households in the site are self-settled refugees, their children are therefore second-generation former refugees. In 2010, schools in the local district were declared amongst the lowest performing in the country [25].

The placement of this study within the Agincourt HDSS provided access to a range of prospectively collected individual and household demographic and SES information on the children, their mothers, and their families. The data used in this study therefore come from two sources: selected individual and household-level data collected by the Agincourt HDSS from 1992–2007 and a large cross-sectional study conducted in 2007 of all the children in grades four and six at 10 of the 28 primary schools in the area chosen by random stratification. Stratification was based on the Provincial Department of Education school performance ratings.

Teachers reported on children’s behavioural and emotional difficulties in English. Children provided self-report measures on their emotional problems (anxiety and depression), symptoms of post-traumatic stress, and perceptions of the school environment. Children’s questionnaires were translated into Shangaan (the local language) and back-translated. A separate pilot study was undertaken prior to data collection on approximately 200 grade 4 and 6 children to facilitate this work. Assessments were conducted in the classrooms by two researchers (MC and TH) who were available to provide any necessary clarification. Demographic and personal information were collected on each child to enable matching of cross-sectional data to household Agincourt HDSS results.

### Measures and their Psychometric Properties

Our principal area of interest was children’s behavioural and emotional adjustment; these were the latent variables that we aimed to assess. Emotional adjustment (internalising state) is best assessed by the child’s own self report while behaviour (externalising state) is best assessed by an external observer such as caregiver or teacher report. While there are no measures specifically validated with cut-off points for this population sample, a number of widely used relevant and validated measures have been used in other parts of Africa. These were the basis of the instruments that were identified for this study.

**Figure 1:**
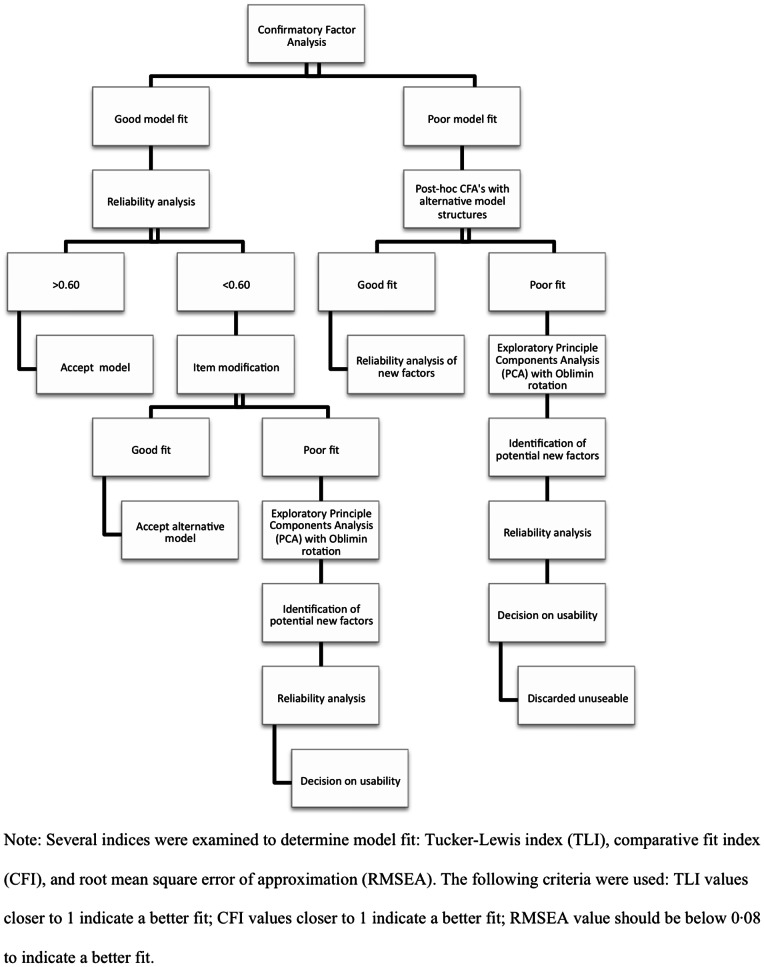
Protocol to determine the psychometric properties of each questionnaire.

The psychometric properties of these scales were examined in detail to ensure their suitability for use in the population. Only scales with good psychometric properties were included in the final analysis (Figure 1). We chose the Strengths and Difficulties Questionnaire (SDQ) because of its wide use internationally and its acceptability to teachers in our pilot work. While the SDQ covers both emotional and behavioural adjustment, the child reports needed to focus more specifically on emotional (internalizing) problems, which is well- addressed by the Youth Self Report (YSR).

#### Teacher-reported scales

Behavioural and emotional problems were assessed with the 25-item child- and teacher-reported SDQ [26]. The SDQ has been shown to be a good screening tool to assess a broad range of symptoms in a population, has been widely used in socioeconomically deprived contexts, and has been validated in several languages [27]–[29]. It has established validity and reliability [26] and assesses a breadth of symptoms in five subscales (conduct problems; emotional symptoms; hyperactivity; peer problems and prosocial behaviour) as well as giving a total difficulties score. The self-report version [30] is generally suitable for children aged around 11–16, depending on their level of literacy and comprehension. More recent studies have shown that it is possible to use this self-report reliably in those 10-years and older [31], [32].

#### Child-reported scales

Anxiety and depression were assessed with the 13-item Youth Self Report (YSR) anxious/depressed scale [33]. The YSR is widely used and targets 11–18 year olds. Although it is normed on a mixed-ethnicity US population, the YSR has been used in many different contexts, including Southern Africa [34]. It has good reliability and validity even when the subscales are used separately [35].

Post-traumatic stress symptoms were assessed with the 44-item Trauma Symptom Checklist for Children –Alternate form (TSCC-A) [36]. The TSCC was selected because it has been shown to be a useful tool for assessing symptoms of chronic trauma and distress and because it evaluates children’s responses to unspecified traumatic events in a number of different symptom domains. It has been standardized on a large sample of racially and economically diverse children from a variety of urban and suburban environments providing norms according to age and sex [36]. Furthermore, dissociative symptoms are measured which have been reported to be common in some sub-Saharan societies [37]. Perception of the school environment was assessed with six questions adapted from a questionnaire assessing social literacy in schools [38]. Items were chosen and adapted from the Peace Zone questionnaire, a US inner-city programme developed to teach social literacy in schools [38]. The questions were administered on a four-point likert-type scale (all of the time, most of the time, sometimes, never), with the instructions: ‘Answer the items for what you are thinking RIGHT NOW’.

Identification of signs of psychopathology in children in South Africa can be challenging, and assessment can be complex. [39] It was therefore important to assess a range of potential difficulties. The SDQ and YSR have been previously found to be highly correlated [40], however as both had not been previous used in the population, we deemed it important for validity to include two similar measures.

#### Translation and back-translation

In line with Parry’s [41] recommendations, this research followed a specific process of translation, back-translation and adjustment to assure linguistic equivalence. Two members of the research team, one of whom has a Master’s degree in mental health and the other a research officer, translated the questionnaires into Shangaan. Both were native to the research area and fluent in Shangaan. Each item was discussed in detail to determine an appropriate translation. A third member of the team who was also fluent in Shangaan back-translated the items.

In order to obtain some index of the relative levels of psychological difficulties experienced by children in this study, we looked for published studies from samples of children with characteristics as similar as possible to the current sample. The comparative group chosen for the SDQ teacher-reported total difficulties score was from a study in the Democratic Republic of Congo [42]. The normative values in the YSR Multicultural supplement classifies cultures into three groups [43]. The closest culture to South Africa included in their multi-cultural norm list was Ethiopia, therefore values specified for Group 2 of the YSR anxious/depressed and somatic scales were used as comparison to this Agincourt sample. The YSR data comes from a regional school-based sample of 681 Ethiopian children aged 11–18 [44]. There are no published norms from a sample similar to Agincourt for the TSCC-A, therefore the normative values from the professional manual were used [36]. The normative sample comes from several studies in the United States, resulting in an ethnically diverse sample of 3,008 children aged 8–16 years. Although the norms are not from the same context, there are similarities.

#### Risk and protective factors

A number of variables considered relevant to this context were extracted from the Agincourt HDSS database: age and sex of household head; household socioeconomic status (SES); household size; the number of childcare grants a household receives (another poverty indicator); whether the child’s mother is alive or deceased and whether the mother resides in the household; period mother has been resident (months); maternal education level; mother’s partnership status; refugee status; whether and duration of child’s breastfeeding; period child has been resident in household (months); exposure of household to death in the previous year; and whether the child has work obligations outside of the home.

#### Statistical analyses

Prevalence rates were examined to identify the level of psychological problems displayed by the children (emotional and behavioural problems, anxiety, and depression) and impressions of the school environment.

Statistical analyses were performed using SPSS (Version 17.0.0). We used the expectation-maximization (EM) algorithm [45], [46] to deal with missing values exceeding the prescribed maximum allowable, using simple imputation techniques with 25 iterations.

As described above, cut-off scores from the relevant test manuals were applied to determine primary prevalence rates for each outcome measure for the entire sample and by gender. Classification for the SDQ scales were based on standard norms [47]. Classification for the YSR scales were based on the group 2, YSR Multicultural supplement norms [43]. Classification of the TSCC-A scale was based on the norms specified in the TSCC manual [36].

Univariable and multivariable analyses were conducted to examine the main effects of gender and grade along with any potential interaction effects (gender and grade were entered as fixed factors). One-sample *t*-tests were used to compare means from this sample to those published elsewhere. Where possible, means from similar samples were applied, otherwise published norms were used.

The variables that were significantly correlated with each scale were used as predictor variables in a multiple regression. A separate regression analysis was conducted for each of the core psychological outcomes. The following selection strategy was used: 1) All variables which were significantly correlated (*p*<0.05) were entered into forward stepwise regressions to identify an optimum model. For each regression model, the psychological domain of interest was entered as the dependent variable; 2) Model diagnostics were examined to check for potential violations of the assumptions of multiple regression; 3) *F* values were examined to determine the model significance; 4) Nested models were compared using *F*-tests (*F* change) to determine whether the nested models showed a significant improvement. The coefficient of determination (*R* ^2^) was examined to determine explanatory strength for each model. Standardized residual plots, histograms, and matrix plots were also examined as model diagnostics [46]. Cronbach’s α was used to assess the reliability of scores [48].

The Agincourt website (http://www.agincourt.co.za/index.php/data/) contains information on accessing the HDSS data, including questionnaires, data dictionaries and metadata associated with the Agincourt HDSS. Survey data, such as those used in these analyses, are archived by the MRC/Wits-Agincourt Unit. Tailored data requests can be made, with data extraction and access dependent on appropriate ethical approvals.

## Results

We obtained data initially from 1,228 children in grades 4 and 6 who were attending the 10 selected schools. Parental consent and child assent was obtained for 85% of all selected children. Eighteen students did not complete the questionnaires to a sufficient degree or were not present on the day of assessment, leaving a final sample of 1,025 children (521 boys and 504 girls), of which 40% were from former refugee households.

### Psychometric Properties

Only questionnaires that we found to have good reliability, validity, and model fit in the study site were included. These included the teacher-reported SDQ total difficulties score (α = 0.75) and prosocial behaviour scale (α = 0.80), the YSR anxious/depressed scale (α = 0.63), and the post-traumatic stress scale TSCC-A (PTS; α = .67). The child-reported SDQ had poor psychometric properties and was excluded from the analyses.

#### Prevalence of Psychological Problems

Teacher reported SDQ scores indicated that 420 children (41.0%, 95% CI of 40.9–41.05; mean (M) = 15.01, standard deviation (SD) = 5.02) had significant emotional and behavioural difficulties and 156 (15.2% CI 15.15–15.25; M = 7.3, SD = 2.47) had significant difficulties with prosocial behaviour.

Child-reported YSR scores indicated that 145 children (14.1%, CI 14.05–14.14; M = 9.98, SD = 4.18) scored in the clinical range on the anxious/depressed scale; while 245 children (23.9%, CI 23.86–23.94; M = 12.73, SD = 5.95) reported significant post-traumatic symptoms on the TSCC-A PTS scale.

Examination of prevalence rates by gender indicated that there were no significant gender differences for any of the domains examined. This sample displayed significantly more psychological problems and negative cognitions than comparable samples and normative data (Table 1).

**Table 1:** Table examining the difference between study findings and comparative samples.

	Outcome measure	Agincourt	Comparator		
		mean	mean	*t*	*p*-value
Boys (*n* = 614)	SDQ Total difficulties	15.14	11.7^a^	15.47	<0.01
	SDQ prosocial behaviour	7.17	6.4^a^	7.18	<0.01
	YSR anxious/depressed	9.82	3.4^b^	34.60	<0.01
	TSCC-A post-traumatic stress	12.56	8.6	15.08	<0.01
Girls (*n* = 579)	SDQ Total difficulties	14.86	10.1^a^	21.54	<0.01
	SDQ prosocial behaviour	7.35	6.7^a^	5.82	<0.001
	YSR anxious/depressed	10.15	5.1^b^	27.56	<0.01
	TSCC-A PTS	12.89	9.5	12.93	<0.01

Note: ^a^Values reported from the Democratic Republic of Congo [42];

b Sample of comparison is Group 2 from the YSR scores for multicultural normative samples [43].

#### Perception of the school environment

**Figure 2:**
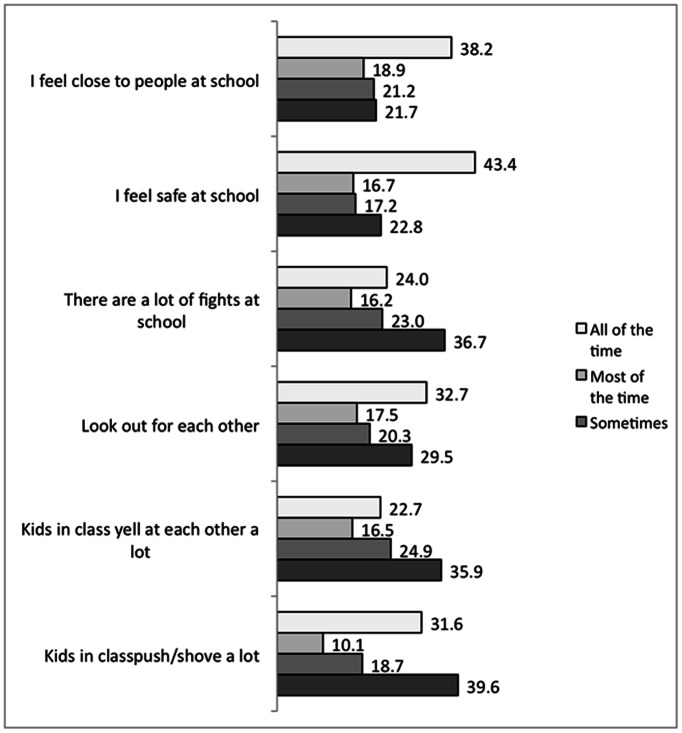
Bar chart depicting children’s perception of their school environment (percent response).

Responses to items regarding children’s perceptions of the school environment are depicted in Figure 2. Nearly 23% reported that they never felt safe at school and 22% reported that they never felt close to people at school.

#### Background risk and protective factors

825 (80%) children in the study were matched to the Agincourt HDSS database to examine socio-demographic and background variables (Table 2).

**Table 2:** Background socio-demographic variables for children in the sample.

*Socio-demographic factors*	*n*	%		*Maternal factors*	*n*	%
**Age of household^+^ head**, n = 779, mean = 50.8 (13.2)				**Mother Status**, n = 822		
**Sex of household head**, n = 779				Deceased	91	11.1
Male	501	64.3		Alive	731	88.9
Female	278	35.7		**Mother Location**, n = 731		
**Number of child grants per household**,				Same household	647	88.5
n = 436, mean = 2.0(1.2)				Elsewhere	84	11.5
1–3	385	88.3		**Mother’s education**, n = 673, mean = 6.1(5.0)		
4–6	49	11.2		0–7 years	386	57.4
7–10	2	0.5		8–15 years	287	42.6
*Child factors*				**Mother’s partnership status**, n = 825		
**Child's grade**, n = 825				Not in partnership	442	53.6
Grade 4	461	55.9		In partnership	383	46.4
Grade 6	364	44.1				
**Sex**, n = 1025						
Male	521	50.8				
Female	504	49.2				
**Second-generation refugee**, n = 824						
Yes	328	39.8				
No	496	60.2				
**Deaths in last year in household**, n = 913						
Yes	70	7.7				
No	843	92.3				

Note: Taken from the Agincourt HDSS 2007. ^+^A household is defined as “a group of people who reside and eat together, plus the linked temporary migrants who would eat with them on return” [62]. Location refers to the physical dwelling place at which an individual is located.

For the teacher-reported SDQ, being from a former refugee household, maternal education, and SES were associated with scores in the univariate analysis; with two of those variables predicting the total difficulties score in multiple regression analyses: being from a former refugee household and lower maternal education level (Table 3). Child’s grade, second-generation refugee status, maternal education level and number of household childcare grants were found to be associated with prosocial behaviour in univariable analyses, however when they were all entered into a multiple regression model, only being from a refugee family and lower child grade significantly predicted difficulties in prosocial (Table 3).

**Table 3:** Multiple regressions for teacher- and child-reported questionnaires identifying risk factors.

Scale	Model		B	std error	*β*	*t*	95.0% CI for coefficient		*F*	*p-value*	*R* ^2^	Δ *R* ^2^
							Lower	Upper				
Total Difficulties Score (*n* = 637)	1	(Constant)	14.14	0.25		56.85	13.65	14.63	22.23	0.00	0.03	0.03
		Child is second-generation refugee	1.89	0.40	0.18	4.71	1.11	2.68				
	2	(Constant)	15.00	0.43		34.68	14.15	15.85	14.16	0.00	0.04	0.01
		Child is second-generation refugee	1.38	0.45	0.18	3.07	0.50	2.27				
		Mother education years	−0.11	0.04	−0.11	2.44	−0.19	−0.02				
Prosocial Behaviour (*n* = 87)	1	(Constant)	7.99	0.30		26.44	7.39	8.60	7.88	0.006	0.09	0.09
		Child is second−generation refugee	−1.50	0.53	−0.29	2.81	−2.56	−0.43				
	2	(Constant)	5.39	1.25		4.33	2.92	7.87	6.43	0.003	0.13	0.05
		Child is second−generation refugee	−1.58	0.52	−0.31	4.33	−2.62	−0.53				
		Child’s grade	0.57	0.26	0.22	2.15	0.04	1.09				
YSR anxious/depressed (*n* = 825)	1	(Constant)	12.6	0.72		17.62	11.20	14.0	13.55	0.00	0.02	0.02
		Child’s grade	−0.53	0.14	−0.13	3.68	−0.81	−0.25				
	2	(Constant)	12.83	0.72		17.83	11.42	14.25	9.63	0.03	0.02	0.01
		Child’s grade	−0.51	0.14	−0.12	3.57	−0.79	−0.23				
		Mother in partnership	−0.68	0.29	−0.08	2.38	−1.24	−0.12				
TSCC-A PTS (*n* = 808)	1	(Constant)	16.01	1.12		14.37	13.82	18.20	8.57	0.003	0.011	0.012
		Child’s grade	−0.67	0.23	−0.11	2.93	−1.11	−0.22				

For the child-reported measures, mother’s partnership status, number of days a child has lived in the household, and child’s grade were associated with anxious/depressed scores in univariate analyses. Two of these variables were found to predict anxious/depression scores; not having a mother in a partnership and the child being in a lower grade (Table 3). Although lower child grade and larger household size were associated with post-traumatic stress (PTS) scores in the univariate analyses, only child’s grade significantly predicted post-traumatic stress scores, with older children having fewer PTS symptoms (Table 3). Overall, mother’s partnership status, mother’s education level, and second-generation refugee status were the most frequent predictors that emerged from the Agincourt HDSS variables across the different domains.

## Discussion

This research attempts to address some of the gaps in the existing evidence-base for child mental health in rural, Southern Africa. Teachers identified high rates of psychological problems, especially behavioural difficulties, such as conduct and attentional problems, while children self-reported moderately high levels of emotional problems. We identified risk factors, such as being a second-generation former refugee, and protective factors, such as the child’s mother being more educated and in a stable partnership.

Teachers rated over 40% of children as having significant difficulties (as indicated by the SDQ total difficulties score), which is high compared to the 10% identified in UK samples [47]. Teachers also reported that 17% had significant difficulties in pro-social behaviour, compared to 13% in the UK [47]. Rates on the child self-report questionnaires were also elevated with 15% scoring in the clinical range on the anxious/depressed scale, and 24% displaying significant post-traumatic stress symptoms; and almost a quarter reported feeling unsafe at school. Boys and girls had similar levels of psychological problems.

In this sample, a number of socio-demographic factors were related to poorer psychological outcomes including: living with a single mother; lower maternal education level; and being from a former refugee family. Former refugee families often have less access to resources because they are not always eligible for social grants and work opportunities due to a lack of official identification papers [49]. The importance of stability for these children was highlighted by the finding that feeling safe at school and in the community was associated with better psychological adjustment.

This study has a number of limitations. All assessments were made by questionnaire and no clinical interview measures were used. This may have led to an over-estimate in the rates of psychological problems. We used establised psychological measures to assess children’s psychological problems. Although, we established the psychometric value of these measures in this setting, we used the established cut-off points derived from other contexts. There were smaller sample sizes for a number of socio-demographic variables as data were not available for some households, despite thorough fieldwork and quality control measures. Although individual and household risk and protective factors were collected prospectively, the psychological data were collected at one time-point; hence, caution needs to be exercised in interpreting causality. In addition, the effect sizes were low to moderate for some of the socio-demographic variables and warrants further investigation [50]. The birth order of children included in the study was not possible to determine. This study also had a number of strengths. It was conducted on a large, representative sample of children from a clearly defined rural population where detailed annual Agincourt HDSS data had been collected over 15 years, enabling the examination of a number of prospectively collected potential risk and protective factors. The research was conducted with strong support from educational authorities, teachers, and communities, which ensured a high response rate and showed that schools can provide a feasible and sustainable setting for research and the development of potential interventions. The Agincourt district, where the research was undertaken, as with many rural areas of Southern Africa, has high levels of poverty, poor resources, and high rates of HIV [20] and migration [51].

Children and adolescents in more socio-economically disadvantaged areas are at increased risk of both poor educational outcomes as well as increased mental health difficulties [52]. Thus, these children experience the double disadvantage of attending inadequately resourced schools and suffering from emotional or behavioural disorders that together are likely to hamper their learning. Those who have experienced a household ‘shock’ (e.g. job loss, theft, death) progress more poorly through school and are less likely to go to secondary school [53] which jeopardizes their personal and economic future development, and this is more common in rural areas [54]. If teachers perceive almost half of those in their classes to have some level of behavioural difficulties (as found here) then the importance of supporting their efforts within the school environment, especially in the context of large class sizes, cannot be underestimated in order to enhance prospects for all their students [55]. Effective interventions in schools could be focused around identifying, referring and supporting the management of emotional and behavioural difficulties. Mental health provisions for children are limited with poor coverage and access in LMICs, thus community- and school-based services can be an effective and sustainable alternative [56], helping to offset some of the economic burden of psychological problems, such as lost production, premature mortality and expenditures on ineffective care.

While resilience was not formally assessed, it should be emphasized that the majority of children did not show significant problems. In fact, they seemed to be well adjusted, with good peer relationships and pro-social functioning and felt safe at school. Given the relatively adverse living conditions, this suggests that many of these children were resilient and managing well. Thus it should be underlined that despite high levels of risk factors, psychological problems only occurred in a minority and it should not be assumed that children are all adversely affected. Understanding the factors that could be contributing to their resilience is important as this could inform interventions.

The public health implications of focusing on children attending schools in these environments are substantial. If these children find their schools safe and supportive environments, they are more likely to continue to attend school, increasing their likelihood of future employment and opportunity. Those with behavioural and emotional problems are at higher risk of school dropout [57] and this is exacerbated by the fact that, in our study, they were more likely to come from homes with mothers in less stable unions or from homes where migration, either circular and ongoing because of current labor, or past, because of organized violence, might be present. In addition, there are important implications for teenage girls in the region who, if they leave school, are ten times more likely to become pregnant compared to those in school and dropping out of school also increases fourfold the likelihood of being HIV positive [58]. Therefore, these children, within a few years, might become the mothers of the next generation and the unresolved problems they have at school might then perpetuate a negative lifecycle [16] that is evinced by poor maternal health and worse outcomes for the physical and psychological health of their children.

This research also provides a feasible and appropriate method of assessing children’s psychological difficulties in the presence of multiple risk factors. Given that essentially little psychological support is currently available to children in the area, these methods can be used to assess specific difficulties and identify vulnerable children so that the scarce resources can be most suitably applied to provide low-cost and sustainable community interventions. Teachers and schools are a well-placed entry point for research and interventions in deprived areas [59].

### Conclusion

An accurate assessment of the health needs of a population is necessary for proficient planning of services, including mental health services and the school curriculum. This study has indicated that in a rural, socioeconomically disadvantaged area of Southern Africa, children exhibited relatively high levels of psychological problems. Teacher-reported rates were very high in the behavioural domain and children self-identified high symptoms of depression and anxiety. The findings suggest that the next generation face significant mental health difficulties in addition to other physical health challenges facing rural populations in South Africa. It is clear from the relative paucity of child and adolescent mental health research in this region that further detailed study is needed, grappling with some of the complex methodological issues inherent in this work and focusing on the resilience of children. What is perhaps most clear is that significant, and usually unaddressed, mental health problems exist in significant numbers in children in rural South Africa, and mental health needs be a critical component of overall health care planning and is an important arena for interventions [60], [61].
